# Supplementary material for: Transsynaptic interactions between IgSF proteins DIP-α and Dpr10 are required for motor neuron targeting specificity
Source: eLife. 2019 Feb 4;8:e42690. doi: 10.7554/eLife.42690 (PMC6391064; doi:10.7554/eLife.42690)
Supplement: Figure 2—source data 1. [file elife-42690-fig2-data1.docx]

**Figure 2—source data**

| Figure 2 | Genotype | Mean | Std. Error | SEM | N (animals/hemisegment) | p-value |
| --- | --- | --- | --- | --- | --- | --- |
| D | *DIP-α-GAL4*/+>EGFP | 0.767 | 0.427 | 0.0551 | 12/60 | n/a |
|  | *DIP-α-GAL4*>EGFP | 0 | 0 | 0 | 10/30 | <0.0001 |
|  | *DIP-α^1-178^* | 0 | 0 | 0 | 18/100 | <0.0001 |
|  | GAL4 control | 0.904 | 0.296 | 0.0305 | 18/96 | n/a |
|  | *DIP-α-GAL4* > *DIP-α* | 0.967 | 0.183 | 0.0333 | 9/56 | 0.8690 |
|  | *DIP-α^1-178^,eve^RN2^-GAL4*> *DIP-α* | 0.897 | 0.306 | 0.0371 | 14/69 | >0.9999 |
| E | UAS-*DIP-α* | 0.771 | 0.424 | 0.0613 | 8/48 | n/a |
|  | *Elav-GAL4*>*DIP-α* | 0 | 0 | 0 | 6/30 | 0.0001 |
|  | *Mef2-GAL*4>*DIP-α* | 0 | 0 | 0 | 9/54 | 0.0001 |
| F | UAS- *DIP-α*-RNAi | 0.9 | 0.305 | 0.0557 | 6/30 | n/a |
|  | *Elav-GAL4* >  UAS-*DIP-α* RNAi | 0.567 | 0.500 | 0.0645 | 12/71 | <0.0001 |
|  | *DIP-α-GAL4* >  UAS-DIPalpha-RNAi | 0.833 | 0.377 | 0.0544 | 10/60 | 0.0008 |
|  | *Mef2-GAL4* >  UAS-*DIP-α*-RNAi | 0.429 | 0.498 | 0.0596 | 10/59 | <0.0001 |
