# Supplementary material for: Transsynaptic interactions between IgSF proteins DIP-α and Dpr10 are required for motor neuron targeting specificity
Source: eLife. 2019 Feb 4;8:e42690. doi: 10.7554/eLife.42690 (PMC6391064; doi:10.7554/eLife.42690)
Supplement: Figure 3—source data 1. [file elife-42690-fig3-data1.docx]

**Figure 3—source data**

| Figure 3-figure supplement 1 | Genotype | Mean | Std. Error | SEM | N (animals/hemisegment) | p-value |
| --- | --- | --- | --- | --- | --- | --- |
| B | Proximal | 0.140 | 0.350 | 0.0464 | 6/57 | n/a |
| B | Distal | 0.929 | 0.257 | 0.0341 | 6/57 | <0.0001 |
