# Supplementary material for: Transsynaptic interactions between IgSF proteins DIP-α and Dpr10 are required for motor neuron targeting specificity
Source: eLife. 2019 Feb 4;8:e42690. doi: 10.7554/eLife.42690 (PMC6391064; doi:10.7554/eLife.42690)
Supplement: Figure 4—source data 1. [file elife-42690-fig4-data1.docx]

**Figure 4—source data**

| Figure 4 | Genotype | Mean | Std. Error | SEM | N (animals/hemisegment) | p-value |
| --- | --- | --- | --- | --- | --- | --- |
| C | *DIP-α-GAL4*/+>EGFP |  |  |  |  |  |
|  | m4 | 0.833 | 0.379 | 0.069 | 12/30 | n/a |
|  | m20 | 0.767 | 0.430 | 0.0785 | 12/30 | n/a |
|  | m19 | 0.833 | 0.379 | 0.069 | 12/30 | n/a |
|  | m18 | 0 | 0 | 0 | 12/30 | n/a |
|  | m3 | 0.9 | 0.305 | 0.0557 | 12/30 | n/a |
|  | m11 | 0 | 0 | 0 | 12/30 | n/a |
|  | m2 | 1 | 0 | 0 | 12/30 | n/a |
|  | m10 | 1 | 0 | 0 | 12/30 | n/a |
|  | m1 | 0.2 | 0.406 | 0.074 | 12/30 | n/a |
|  | m9 | 0.966 | 0.182 | 0.033 | 12/30 | n/a |
|  | *DIP-α-GAL4*>EGFP |  |  |  |  |  |
|  | m4 | 0 | 0 | 0 | 16/30 | n/a |
|  | m20 | 0.033 | 0.183 | 0.0333 | 16/30 | n/a |
|  | m19 | 0.067 | 0.254 | 0.0463 | 16/30 | n/a |
|  | m18 | 0.067 | 0.254 | 0.0463 | 16/30 | n/a |
|  | m3 | 0.3 | 0.466 | 0.085 | 16/30 | n/a |
|  | m11 | 0.167 | 0.379 | 0.069 | 16/30 | n/a |
|  | m2 | 1 | 0 | 0 | 16/30 | n/a |
|  | m10 | 1 | 0 | 0 | 16/30 | n/a |
|  | m1 | 0.8 | 0.407 | 0.0743 | 16/30 | n/a |
|  | m9 | 0.9 | 0.305 | 0.0557 | 16/30 | n/a |
| Figure 4-figure supplement 1 |  |  |  |  |  |  |
| A | *DIP-α-GAL4*/+>EGFP | 1.407 | 0.659 | 0.0897 | 13/54 | n/a |
| A | *DIP-α-GAL4*>EGFP | 2.746 | 0.882 | 0.1149 | 15/59 | <0.0001 |
|  |  |  |  |  |  |  |
