# Supplementary material for: Transsynaptic interactions between IgSF proteins DIP-α and Dpr10 are required for motor neuron targeting specificity
Source: eLife. 2019 Feb 4;8:e42690. doi: 10.7554/eLife.42690 (PMC6391064; doi:10.7554/eLife.42690)
Supplement: Figure 6—source data 1. [file elife-42690-fig6-data1.docx]

**Figure 6-source data**

| Figure 6 | Genotype | Mean | Std. Error | SEM | N (animals/hemisegment) | p-value |
| --- | --- | --- | --- | --- | --- | --- |
| D | *w^1118^* | 0.764 | 0.429 | 0.058 | 8/62 | n/a |
|  | *dpr6-GAL4/Df* | 0.886 | 0.32 | 0.036 | 12/70 | <0.0001 |
|  | *dpr10^14-5^/Df* | 0.035 | 0.184 | 0.024 | 11/60 | <0.0001 |
| E | UAS-Dpr10-V5 | 0.833 | 0.377 | 0.0544 | 8/48 | n/a |
|  | *Elav-GAL4*>Dpr10-V5 | 0.083 | 0.280 | 0.047 | 6/36 | 0.0001 |
|  | *DIP-α-GAL4*/+>Dpr10-V5 | 0.067 | 0.254 | 0.046 | 5/30 | 0.0001 |
|  | *Mef2-GAL4*>Dpr10-V5 | 0.304 | 0.465 | 0.069 | 8/47 | 0.0001 |
| F | UAS-*dpr10*-RNAi | 0.854 | 0.357 | 0.052 | 8/48 | n/a |
|  | *Elav-GAL4*>*dpr10*-RNAi | 0.329 | 0.473 | 0.057 | 8/48 | <0.0001 |
|  | *DIP-α-GAL4*/+>  *dpr10*-RNAi | 0.767 | 0.427 | 0.055 | 10/60 | 0.5669 |
|  | *Mef2-GAL4*>*dpr10*-RNAi | 0.021 | 0.144 | 0.021 | 8/48 | <0.0001 |
| Figure 6-figure supplement 1 |  |  |  |  |  |  |
| A | (m2) *w^1118^* | 0.966 | 0.182 | 0.0333 | 7/32 | n/a |
|  | (m2) *dpr10^14-5^/Df* | 0.983 | 0.127 | 0.0161 | 7/30 | ns |
|  | (m10) *w^1118^* | 0.966 | 0.182 | 0.0333 | 7/32 | n/a |
|  | (m10) *dpr10^14-5^/Df* | 0.883 | 0.323 | 0.0417 | 7/30 | ns |
|  | (m1) *w^1118^* | 0.142 | 0.356 | 0.0673 | 7/32 | n/a |
|  | (m1) *dpr10^14-5^/Df* | 0.232 | 0.427 | 0.0651 | 7/30 | ns |
|  | (m9) *w^1118^* | 0.9 | 0.305 | 0.0557 | 7/32 | n/a |
|  | (m9) *dpr10^14-5^/Df* | 0.596 | 0.495 | 0.0687 | 7/30 | 0.0018 |
| B | UAS-Dpr10-V5/+ | 1.25 | 0.915 | 0.1619 | 6/30 | n/a |
|  | *Mef2-GAL4*>Dpr10-V5 | 2.833 | 1.621 | 0.2959 | 12/62 | <0.0001 |
