# Supplementary material for: Transsynaptic interactions between IgSF proteins DIP-α and Dpr10 are required for motor neuron targeting specificity
Source: eLife. 2019 Feb 4;8:e42690. doi: 10.7554/eLife.42690 (PMC6391064; doi:10.7554/eLife.42690)
Supplement: Figure 7—source data 1. [file elife-42690-fig7-data1.docx]

**Figure 7-source data**

| Figure 7 | Genotype | Mean | Std. Error | SEM | N (animals/hemisegment) | p-value |
| --- | --- | --- | --- | --- | --- | --- |
| G | Late st14/Early st15 |  |  |  |  |  |
|  | m17 | 0.286 | 0.488 | 0.184 | 7 | n/a |
|  | m16 | 0 | 0 | 0 | 7 | n/a |
|  | m15 | 0 | 0 | 0 | 7 | n/a |
|  | m29 | 0 | 0 | 0 | 7 | n/a |
|  | m28 | 0 | 0 | 0 | 7 | n/a |
|  | m27 | 0 | 0 | 0 | 7 | n/a |
|  | m26 | 0 | 0 | 0 | 7 | n/a |
|  | m25 | 0 | 0 | 0 | 7 | n/a |
|  | m7 | 0 | 0 | 0 | 7 | n/a |
|  | m6 | 0 | 0 | 0 | 7 | n/a |
|  | m30 | 0 | 0 | 0 | 7 | n/a |
|  | m14 | 0 | 0 | 0 | 7 | n/a |
|  | m13 | 0 | 0 | 0 | 7 | n/a |
|  | m12 | 0 | 0 | 0 | 7 | n/a |
|  | m5 | 0.571 | 0.535 | 0.202 | 7 | n/a |
|  | m8 | 0 | 0 | 0 | 7 | n/a |
|  | m21 | 0 | 0 | 0 | 7 | n/a |
|  | m22 | 0 | 0 | 0 | 7 | n/a |
|  | m23 | 0 | 0 | 0 | 7 | n/a |
|  | m24 | 0 | 0 | 0 | 7 | n/a |
|  | m4 | 0 | 0 | 0 | 7 | n/a |
|  | m20 | 0.571 | 0.535 | 0.202 | 7 | n/a |
|  | m19 | 0 | 0 | 0 | 7 | n/a |
|  | m18 | 0 | 0 | 0 | 7 | n/a |
|  | m3 | 0 | 0 | 0 | 7 | n/a |
|  | m11 | 0 | 0 | 0 | 7 | n/a |
|  | m2 | 0 | 0 | 0 | 7 | n/a |
|  | m10 | 0 | 0 | 0 | 7 | n/a |
|  | m1 | 0 | 0 | 0 | 7 | n/a |
|  | m9 | 0 | 0 | 0 | 7 | n/a |
|  | Late st15/Early st16 |  |  |  |  |  |
|  | m17 | 0.5 | 0.548 | 0.224 | 6 | n/a |
|  | m16 | 0 | 0 | 0 | 6 | n/a |
|  | m15 | 0.5 | 0.548 | 0.224 | 6 | n/a |
|  | m29 | 0.333 | 0.516 | 0.211 | 6 | n/a |
|  | m28 | 0 | 0 | 0 | 6 | n/a |
|  | m27 | 0.5 | 0.548 | 0.224 | 6 | n/a |
|  | m26 | 0 | 0 | 0 | 6 | n/a |
|  | m25 | 0 | 0 | 0 | 6 | n/a |
|  | m7 | 0 | 0 | 0 | 6 | n/a |
|  | m6 | 0 | 0 | 0 | 6 | n/a |
|  | m30 | 0 | 0 | 0 | 6 | n/a |
|  | m14 | 0 | 0 | 0 | 6 | n/a |
|  | m13 | 0 | 0 | 0 | 6 | n/a |
|  | m12 | 0 | 0 | 0 | 6 | n/a |
|  | m5 | 0.667 | 0.516 | 0.211 | 6 | n/a |
|  | m8 | 0 | 0 | 0 | 6 | n/a |
|  | m21 | 0 | 0 | 0 | 6 | n/a |
|  | m22 | 0 | 0 | 0 | 6 | n/a |
|  | m23 | 0 | 0 | 0 | 6 | n/a |
|  | m24 | 0 | 0 | 0 | 6 | n/a |
|  | m4 | 0 | 0 | 0 | 6 | n/a |
|  | m20 | 1 | 0 | 0 | 6 | n/a |
|  | m19 | 0 | 0 | 0 | 6 | n/a |
|  | m18 | 0 | 0 | 0 | 6 | n/a |
|  | m3 | 0 | 0 | 0 | 6 | n/a |
|  | m11 | 0 | 0 | 0 | 6 | n/a |
|  | m2 | 0.333 | 0.516 | 0.211 | 6 | n/a |
|  | m10 | 0 | 0 | 0 | 6 | n/a |
|  | m1 | 0 | 0 | 0 | 6 | n/a |
|  | m9 | 0 | 0 | 0 | 6 | n/a |
|  | Late st16/Early st17 |  |  |  |  |  |
|  | m17 | 0.875 | 0.354 | 0.125 | 8 | n/a |
|  | m16 | 0.125 | 0.354 | 0.125 | 8 | n/a |
|  | m15 | 0.75 | 0.463 | 0.1637 | 8 | n/a |
|  | m29 | 1 | 0 | 0 | 8 | n/a |
|  | m28 | 0.375 | 0.518 | 0.183 | 8 | n/a |
|  | m27 | 0.875 | 0.354 | 0.125 | 8 | n/a |
|  | m26 | 0.375 | 0.518 | 0.183 | 8 | n/a |
|  | m25 | 0.125 | 0.354 | 0.125 | 8 | n/a |
|  | m7 | 0.125 | 0.354 | 0.125 | 8 | n/a |
|  | m6 | 0.25 | 0.463 | 0.164 | 8 | n/a |
|  | m30 | 0.375 | 0.518 | 0.183 | 8 | n/a |
|  | m14 | 0.5 | 0.535 | 0.189 | 8 | n/a |
|  | m13 | 0.75 | 0.463 | 0.1638 | 8 | n/a |
|  | m12 | 0.75 | 0.463 | 0.164 | 8 | n/a |
|  | m5 | 0.875 | 0.354 | 0.125 | 8 | n/a |
|  | m8 | 0 | 0 | 0 | 8 | n/a |
|  | m21 | 0 | 0 | 0 | 8 | n/a |
|  | m22 | 0 | 0 | 0 | 8 | n/a |
|  | m23 | 0 | 0 | 0 | 8 | n/a |
|  | m24 | 0 | 0 | 0 | 8 | n/a |
|  | m4 | 0.25 | 0.463 | 0.164 | 8 | n/a |
|  | m20 | 1 | 0 | 0 | 8 | n/a |
|  | m19 | 0.125 | 0.354 | 0.125 | 8 | n/a |
|  | m18 | 0 | 0 | 0 | 8 | n/a |
|  | m3 | 0.714 | 0.488 | 0.184 | 8 | n/a |
|  | m11 | 0 | 0 | 0 | 8 | n/a |
|  | m2 | 0.857 | 0.378 | 0.143 | 8 | n/a |
|  | m10 | 0.429 | 0.535 | 0.202 | 8 | n/a |
|  | m1 | 0.429 | 0.535 | 0.202 | 8 | n/a |
|  | m9 | 0.429 | 0.535 | 0.202 | 8 | n/a |
|  |  |  |  |  |  |  |
